# A Privacy-Preserving Desk Sensor for Monitoring Healthy Movement Breaks in Smart Office Environments with the Internet of Things

**DOI:** 10.3390/s23042229

**Published:** 2023-02-16

**Authors:** Ananda Maiti, Anjia Ye, Matthew Schmidt, Scott Pedersen

**Affiliations:** 1 School of ICT, CoSE, University of Tasmania, Launceston, TAS 7248, Australia; 2 Active Work Laboratory, CALE, University of Tasmania, Launceston, TAS 7248, Australia; 3 School of Health Sciences, CoHM, University of Tasmania, Launceston, TAS 7248, Australia

**Keywords:** privacy-preserving, privacy, time series, smart office, smart building, Internet of Things, microcontroller, eHealth, sedentary behavior, activity recognition

## Abstract

Smart workplace Internet of Things (IoT) solutions rely on several sensors deployed efficiently in the workplace environment to collect accurate data to meet system goals. A vital issue for these sensor-based IoT solutions is privacy. Ideally, the occupants must be monitored discreetly, and the strategies for maintaining privacy are dependent on the nature of the data required. This paper proposes a new sensor design approach for IoT solutions in the workplace that protects occupants’ privacy. We focus on a novel sensor that autonomously detects and captures human movements in the office to monitor a person’s sedentary behavior. The sensor guides an eHealth solution that uses continuous feedback about desk behaviors to prompt healthy movement breaks for seated workers. The proposed sensor and its privacy-preserving characteristics can enhance the eHealth solution system’s performance. Compared to self-reporting, intrusive, and other data collection techniques, this sensor can collect the information reliably and timely. We also present the data analysis specific to this new sensor that measures two physical distance parameters in real-time and uses their difference to determine human actions. This architecture aims to collect precise data at the sensor design level rather than to protect privacy during the data analysis phase.

## 1. Introduction

The Internet of things (IoT) is increasingly being deployed in smart building environments for various applications such as internal ventilation and human activity monitoring. A recent trend in this regard is the monitoring of the sedentary behavior of employees or people for health purposes [1]. The nature of workplace activities and health issues has changed over the last few decades due to advancements in computing and networking technologies. This has resulted in prolonged sitting for employees, which can lead to negative, long-term health impacts [2].

Workplace health *intervention* mechanisms are designed to improve the health of desk-based workers by increasing their total daily movement, decreasing their net sedentary time, and breaking up prolonged periods of sedentary time. This has become the focus of a significant amount of health prevention research in the 21st century [3]. There is a growing interest in tracking postures and movement in office environments [4,5,6,7,8]. Some of these have been implemented as full-fledged eHealth applications [9].

eHealth solutions can be developed to nudge employees to engage in healthy behavior through prompting reminders on desktops or personal mobile devices. For example, a digital eHealth solution developed in Tasmania, Australia, named Exertime^TM^, works by prompting employees to take regular, short-duration, low-intensity movement breaks throughout the workday. These prompts allow employees to choose from a set of office-appropriate exercises and record their performance [10]. These eHealth solutions, when implemented in offices, can become an integral part of the intelligent environment and *smart building* solutions.

Workplace prompting software (e.g., Exertime^TM^) may provide a suitable prevention strategy for desk-based employees to improve their physical health (e.g., decreased blood pressure [11]), and mental health [12]. Putatively, these health improvements are caused by the Exertime^TM^ prompts changing a person’s desk-based behavior, i.e., the amount and pattern of sedentary behavior and movement while in the workplace. However, no empirical evidence supports this hypothesis, as the employee’s movement and sedentary behavior were *not* measured objectively. Instead, self-reported measures of adherence to the program were used as a proxy for direct measurements of behavioral change [9,10,13,14,15,16,17]. Many (e.g., [18,19]) have criticized the inaccuracy and bias of self-reported data to recall sedentary behavior in the workplace. Thus, with no quantitative measure of workplace behavior, the cause of improvements in health associated with workplace prompting software (e.g., Exertime^TM^) cannot be determined. The observed changes in health may be due to changes in movement patterns during the workday or other confounding factors related to the intervention.

Thus, in this paper, we propose and validate an inconspicuous, low-cost, desk-based sensor to quantitatively measure employees’ on-desk behavior and the initiation of movement breaks prompted by the software. Desk-based behavior is defined as the amount and pattern of time a person is (1) sitting or (2) standing at their desk, and (3) the time spent away from it. The development of these sensors will measure these three desk behaviors to enable “smarter” prompting from the IoT system to the employees. This will allow future, larger-scale studies to quantify the changes in desk-based behavior elicited by eHealth prompting software and relate program adherence and behavioral changes to the observed improvements in health.

The IoT has the ability to collect information from embedded devices in the environment and to process these data to achieve desired goals. Such internet and cloud-connected devices hold great promise for improved smart health care solutions [20]. Here, we propose a novel, specially designed IoT sensor that can be plugged into the user’s monitor and communicates with a server to determine, in real time, user changes in posture, e.g., *sitting* or *standing* events, and their presence at their desk. Several prior works have attempted to measure workplace movement [4,6,7,8,21,22,23,24,25,26,27]. These technologies are at different stages of development. However, all have issues that prevent their wide-scale adoption in an eHealth intervention:

- Scalability issues in terms of cost and range;
- Collective monitoring which lacks specific user details;
- Use of an environmental factor that is not guaranteed in all workplace environments;
- Prohibitive cost;
- Does not meet the privacy standards of all workplaces.

The critical factor in designing the sensor is privacy, as the application aims to track human behavior in the workplace. Thus, the system cannot capture information that might have legal implications or is unethical. It is assumed that the employees accept the minimal tracking of their movement to ensure proper movement breaks to receive health benefits. However, it must be guaranteed to the employees that no other data, such as *conversation* or *attendance,* are also captured.

Privacy-preserving techniques have been widely used for machine learning and data analytics once the data are collected in various applications [28]. Such an approach often relies on the programmatic anonymization of data sets before they can be processed [29]. Other techniques involve data encryption to transmit and store data securely, such as in the case of social media data [30,31]. While this is acceptable and unavoidable in some high-risk applications, we aim to provide a design framework for a device with a data collection strategy that collects minimal data, resulting in smaller, application-specific data sets. This strategy is expected to be vital in making the low-risk application widespread, with a higher user confidence and uptake. Hence, this paper focuses on streamlined data collection at the sensor level that preserves privacy.

The contributions of this paper include a framework for designing privacy-preserving, sensor-based IoT solutions. Several key characteristics of implementing a privacy-preserving sensor can be generalized to multiple applications. The second contribution is the design of a specific, low-cost sensor for the purpose of detecting two specific human movements in the office: standing up and sitting down. This sensor is intended to be used for an eHealth application to identify the actions and prompt when necessary. A detailed discussion of integrating the sensor in a cloud-based platform is also provided.

The paper is organized as follows: Section 2 presents the related work regarding sensing strategies in office environments and time series analysis. Section 3 presents the new, privacy-preserving sensor design framework and the new sensor design and data analysis technique. Section 4 presents the implementation and results of a prototype sensor, followed by results in Section 5 and suggestions for future work (Section 6) and conclusions (Section 7).

## 2. Related Work

### 2.1. Human Exercise in an Office Environment

Uninterrupted, prolonged sitting during work hours adversely affects employees’ physical and mental health. While widespread, the adoption of standing desks has been shown to be ineffective in ameliorating this health hazard [32]. Our previous research has suggested that movement breaks away from the desk during the workday can improve physical [15] and mental health [12]. How these healthy movements in the field can be measured is less certain. Naturally, a camera faced at each seated employee could solve this measurement problem. However, the surveillance and privacy concerns of this technology being used during field-based research prevail [33]. Thus, many public health researchers have turned to self-reporting measures (e.g., questionnaires) of sedentary and movement behavior in the workplace. However, the validity and reliability of these tools are less than desirable. For example, although diaries and logs may be more reliant than questionnaires because of their reliance on shorter recall periods, a higher associated cost burden may result in lower uptake and compliance rates [34].

Thus, a better solution is warranted to measure desk occupancy and, more importantly, how often employees interrupt their seated posture with standing to engage in a movement break during the workday. A current meta-analysis [35] suggests that seated employees should stand to take a movement break at least once every two hours during the workday to avoid vascular malfunction.

The existing strategy of the eHealth system is to prompt the user with a reminder to move at specific points in time during office hours. There are specific settings for the gap between the timed prompts, and these are dependent on the user and are set in hours. The current system cannot identify the users’ urgency to complete their work or pay attention to the prompt. Also, the user has to self-report their movement, and the software does not have any way to confirm any activity—neither in response to the prompt nor during the gap before it.

The aim of the proposed system is to keep continuous track of the user’s movement and identify if they moved, i.e., stood up and sat down, even when they were not prompted. If the system can positively identify this action, it can confirm that the movement break has occurred within the time gap. If the system has detected movement but is not 100% certain of the action, it can ask the user to perform the action and monitor it upon prompt.

### 2.2. Sensing Strategies in Office Environments

An essential characteristic of the eHealth system is that the data collected can be sensitive and must be stored securely and encrypted during transmission [36]. Sensing in an office usually follows two approaches:

- Use an *individual* sensor per person in the office. This way, the system can track the individual person in the system and measure their activities. This creates the risk of privacy invasion;
- Use an *environmental* approach, in which sensors are placed in a global setting to observe all persons in the office. This approach can collect collective information, and it can be verified whether a certain degree of movement or other activities have happened in the office. It represents the actions of all people but cannot make any decision specific to one person.

A number of sensor modalities are or can be used in the workplace to assess worker movements:

- *Accelerometry*

Traditionally, wearable sensors, such as those developed by companies such as PAL Technology and Fitbit, are becoming increasingly popular in the workplace to track and improve individual sedentary behavior. These sensors are typically worn on the wrist or around the waist and use advanced technology to monitor and measure various aspects of an individual’s physical activity, including the time spent seated, standing, and sitting–standing transitions. By providing objective measurements of an individual’s sedentary behavior, these sensors can help to encourage more active lifestyles and reduce the risk of health issues associated with prolonged periods of sitting such as obesity, hypertension, and diabetes. Overall, wearable sensors represent a powerful tool for measuring sedentary behavior and improving well-being in the workplace. However, a systematic review [37] on mobile health intervention solutions revealed several barriers to using wearable technology in field-based research. Two of these studies reported broken or lost devices [38,39], and another two studies indicated a lack of interest by the participants [40,41], as well as several fashion, technical, and usability concerns [41].

- *Camera*

Using cameras in the workplace to monitor employee compliance with workplace health interventions is considered a gold standard measurement for researchers [33]. However, employees often express concerns about surveillance and privacy when participating in field-based studies [42,43,44,45]. There are ethical concerns as the camera can record more information than the proposed system.

- *Passive Infrared (PIR) sensors*

PIR is commonly used in office environments to determine the presence of a person in the area within view of the sensor. These sensors detect changes in infrared radiation emitted by all objects. When an individual enters a room, their body heat will cause a difference in the infrared radiation, triggering the PIR sensor. This information can then be used for tracking employee presence without recording individual identity [46]. PIR sensors are highly accurate and efficient, making them a popular choice for the workplace [47]. However, a PIR sensor has limitations. It can only detect a person’s presence, and it returns binary data, which are not detailed enough to assess multiple people and movements.

- *Pressure sensor*

Pressure sensors could also be attached to chairs to gather information about a person sitting and the number of occupants sitting on chairs with high accuracy usage data [48]. However, when pressure sensors are installed on chairs, there is a significant limitation as a standing employee cannot be detected. Moreover, seat pads are conspicuous during data collection, alerting participants to unwanted and wanted behaviors.

- *Sound sensor*

Sound sensors, also known as microphones, detect and measure sound waves. Sound sensors have also been utilized in the field of human behavior recognition [49,50,51]. Acoustic data can be categorized into several behavioral patterns [49]. These may include encouraging regular breaks during meetings or conference calls or encouraging individuals to stand or walk around while using the microphone. However, similar to cameras, microphones can record more information than the minimum requirement causing surveillance concerns.

Researchers have used body-worn sensors and desk/chair-based sensors to measure sedentary behavior at work. However, they are not appropriate for future long-term studies of desk-based behavior due to their limited battery life, the need for participant interaction, and the reactivity of the participant to a conspicuous sensor. The most common body-worn sensor researchers use is the thigh-worn accelerometer, which is taped to the participants’ anterior thigh and is able to both measure the participant’s step count and differentiate between seated and standing postures (e.g., [52]). Seat-based sensors have also been used to quantitatively assess desk-based employees’ movement [48,53,54]. These authors utilized a large, conspicuous, instrumented seat pad to measure and record the temporal aspects of sedentary behavior in the workplace over a typical workday. The body-worn sensor battery life is limited to seven days of recording time, which is negligible compared to a future trial that may last up to a year in duration. Additionally, the body-worn sensor and the seat pad constantly reminded participants that their movements were being measured in the field. This could affect the unwanted (sitting) and wanted (standing for a movement break) behaviors, introducing an unwanted Hawthorne effect.

Thus, this work aims to develop and validate inconspicuous, non-wearable sensors to measure office-based behavior, thereby supporting future studies of desk-based behavior eHealth interventions such as Exertime^TM^.

### 2.3. Time Series Classification

The device in the proposed system collects two sets of distance data in real time. This generates two time series. A time series is a set of points accumulated at equal intervals of time. The interval is determined by the sampling rate of the device. Often, time series iare collected in real time and must be processed in real time as well.

Time series analysis and classification involve identifying a sub-sequence of time series that closely matches a known pattern. Time series analysis is used extensively in speech analysis, in which the software tries to match the spoken word in audio to a known pattern of speaking the word.

Several solutions exist for performing this analysis and classification, such as dynamic time warping (DTW) [55,56], BOSS [57], and the time series forest method [58]. Regardless of the time series analysis method, the algorithm uses a cost function (e.g., *alignment cost*) to determine the similarity between the incoming data and a known pattern of data that reflects the behavior we wish to classify. If the alignment cost exceeds a threshold, then that behavior is recognized. The key challenge to these methods is that the algorithm needs to account for the behavior being performed faster or slower or with varying amplitude when compared to the known data pattern.

A critical decision in choosing a time series classification technique is whether the classification needs to happen in real time with incoming data or offline with prerecorded information. In the proposed application in this paper, we use the DTW method, which can support a high sampling rate of incoming information.

## 3. Methodology—The Proposed Architecture

This section describes the sensor, its privacy-related issues, and the data analysis strategies used. The method of integrating the sensors with a cloud-based eHealth IoT system is also addressed.

### 3.1. Privacy Preservation vs. Monitoring

Privacy is an essential issue in every human-related computing system. The level of privacy needed can be easily established if the environment is governed by individuals, such as in smart home systems. The default approach is to use a common sensor for multiple applications, e.g., cameras. However, in smart office systems, the environment does not belong to any single person, and it is virtually impossible to set a universal set of privacy parameters acceptable to everybody.

Any application that uses the IoT as a solution intends to monitor several variable aspects of human operation. The aim is always to identify specific *events* with respect to time. There is an acceptable threshold between preserving privacy and monitoring the variables of interest. For a system that needs continuous feedback on human actions, it is necessary to not capture information irrelevant to identifying those events.

The keys to implementing a privacy-preserving sensor are:

- Identifying the exact minimum parameter of the target human actions. This can be accomplished with the specific sensor associated with the parameter. Using cheaper components increases the risk of noisy data. However, modern data analysis and machine learning techniques can mitigate this problem. A vital disadvantage of privacy-preserving sensors is their scalability across multiple applications. With the sensor being highly customized for the application to *not* capture unnecessary information, it becomes difficult to use the same device for various applications. In a fixed environment, it may be difficult to implement numerous devices per human user;
- Keeping the device form factor small and low in cost. The devices should have sufficient computing power by themselves to collect the necessary information and put minimal workload on the user’s computer in the office.

In the context of a movement tracking sensor, it is important not to capture additional information aside from the targeted movements. This means the best outcome cannot involve capturing video, audio, or keyboard inputs. These always have additional information that cannot be recorded or processed in most office-based IoT applications.

### 3.2. Privacy-Preserving Sensing

The features that are typically measured in an office environment are human movements. Sensing for human-related information in an office environment is constrained and influenced by several factors. The following three parameters are vital to designing a privacy-preserving sensor:

*i*. *Precise Information*: In most office environments, individual users must not be identifiable in the overall system using the sensor system. This is a critical requirement as most office workers will not consent for their information, such as video or audio recordings, to be collected and stored. This implies that the sensor system and the corresponding software solution must be privacy-preserving. In many instances, this rules out devices that are most common and can easily monitor the worker, e.g., cameras and microphones;
*ii*. *Traceable*: For proper, automated prompting of an individualized eHealth solution, the software must keep track of individual people in the system for an extended period. Tracking in this context is simply knowing whether a particular person has moved or not. No other activities must be collected. Tracking also enables a direct feedback mechanism. In the proposed system, to prompt the employees in real time, the prompts must be generated according to the individual person’s actions and health behaviors;
*iii*. *Isolated event detection or managing crowded environments*: In crowded environments, which offices typically have, multiple employees will be working within the same environment. In such a case, multiple employees may be close to each other and within the radius of the sensor. The sensor must be able to detect the target person only in this environment. This requirement rules out most PIR sensors as they have a static range that makes it impossible to identify an employee uniquely in order to enable individualized prompts.

**Figure 1:**
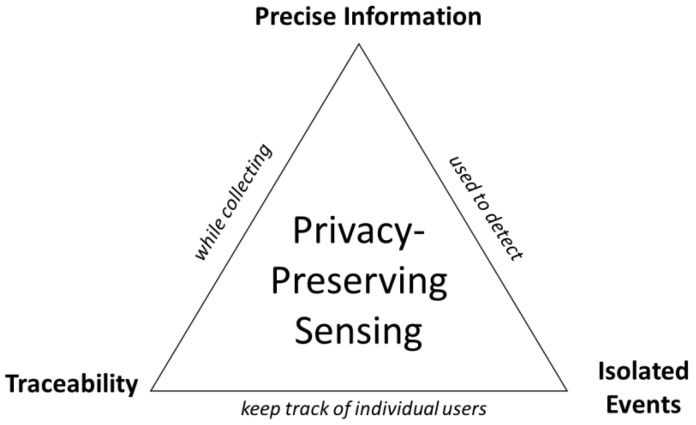
The privacy-preserving triad of features.

Figure 1 shows the relation between the three features. Apart from these key features, a privacy-preserving sensor should have the features following as well:

*iv*. *Comfort:* The devices deployed must not interfere with the work of the people in the office. A critical decision for researchers and developers is to use *wearable* or *non-wearable* sensing systems. Wearable sensors work by placing small devices on the employees during office hours. Using wearable sensors can cause distractions, and the data collected by them can be impacted due to improper fittings. The non-wearable sensors, which are privacy-preserving, typically have limitations on how much data they can collect in the office space;
*v*. *Security:* The software solution may pose a cyber security risk if the sensor requires communication with the workers’ computers. Even if the software solution itself is trusted, it becomes an administrative challenge to selectively deploy permissions for the software solutions. This is an issue with tracking keyboards and mouse movement in some office environments;
*vi*. *Cost*: The devices, which must be small and fit within the workspace seamlessly, must have a low cost-per-worker ratio;
*vii*. *Energy consumption*: The devices should be powered easily and have a small power consumption even if they work in real time.

Table 1 presents a detailed comparison of various technologies used to monitor workplace behavior.

While many of the solutions presented here can maintain privacy, they do not collect enough information for traceability or isolated event detection, reducing the overall effectiveness of the devices for long-term health benefits. Our proposed device is in the form of a USB peripheral that can monitor the movements of one person sitting at their desk. The challenge in detecting these movements are:

- Designating the difference between standing up and sitting at the desk;
- There is a defined radius in which the system must look for the user as there may be another person in the same room;
- It must work with modern office work styles. For example, due to an increase in online meetings, people may not use keyboards and mice for extended periods of time, and timeouts cannot be imposed on such input devices;
- Real-time detection of movement that enables appropriate prompting by the eHealth solution on a timely basis. If the sit–stand action is confirmed within a time period, then there is no prompt or intervention.

### 3.3. The Sensor Device

The proposed sensing architecture comprises a software client and a physical hardware sensor installed at each individual desk in the office. The physical sensor consists of at least two *distance sensor*s and one *tilt sensor*. The orientation of the sensors inside the physical device is permanently fixed, but the whole sensor device can be adjusted for each installation. The sensor is attached to the monitor bezel and can be positioned on the top or bottom of the monitor.

### 3.4. The Differential Distance Design

The proposed differential design architecture is based on continuously measuring two distances (*d_b_* and *d_a_*). The distance can be reliably measured with several cost-effective sensors that are readily available off the shelf. Sensor *B* is placed such that it points just over the edge of the desk into the person’s torso. This distance is *d_b_*. Typically, due to ergonomics and table sizes, this distance is expected to be

80 cm *< d_b_ <* 100 cm

Sensor *A* is aligned on top of sensor *B*, pointing just over the sitting height of the person at the desk. While people are of different heights, there is less variation in height between people while they are seated. The device has an adjustable position of both the sensors in a *vertical* direction, which alters the angle *θ* to accommodate a range of a desk and person characteristics. Considering the required ergonomic settings in modern offices, the impact of the angle on the accuracy of detecting movement events would be minimal. However, when installing the device, users can be advised on proper installations regarding the best range of *θ* for a desk and user.

**Figure 2:**
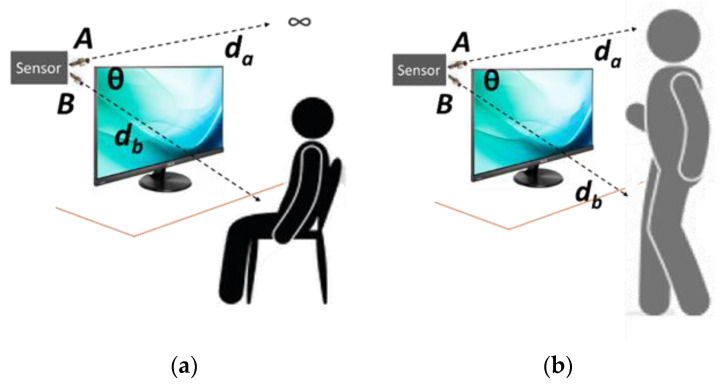
The sensor design and installation are set up (**a**) when the person is seated and (**b**) when the person is standing.

Figure 2 depicts the device setup. It is assumed that workers typically sit at their desks in the office. The proposed system then calculates the difference in the *d_a_* and *d_b_* in real time.

**Figure 3:**
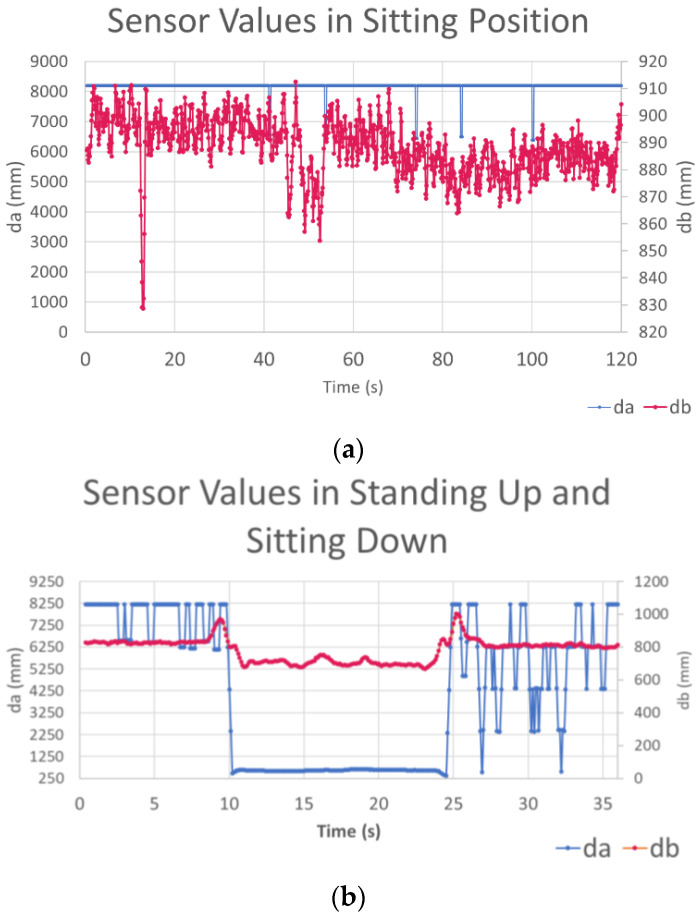
The typical raw time series data from *d_a_* and *d_b_* in real-time. The expected data pattern when sitting down and standing up is shown in (**b**). (**a**) Displays the data when the person is sitting continuously.

Figure 3 shows the default patterns of time series data obtained from the sensor in two different situations. These data were collected at 10 Hz and presented as filtered, with a moving average of a window size of four. The first position in Figure 3a is when the person is sitting at the desk. Due to natural human upper body movements when seated, there is noise in the data during these periods in *d_b_*. The *d_a_*, on the other hand, is at an infinite distance as it is directed above the seated person’s head. In this case, the sensor returns a maximum distance of 8190 mm.

Figure 3b shows the time series data collected from a seated person standing up and then sitting down. The person does not move away from the desk. There is a distinct pattern in *d_b_* when the person stands up and then sits between 8 and 12 s and 24 and 27 s. The value for *d_a_* drops to 250 mm as the person remains standing.

A *tilt sensor* ensures that the *d_a_* keeps pointing above the head of the user. If, due to any shaking, the sensor’s orientation changes, the tilt sensor will detect that the configuration has altered. This is necessary as the *d_a_* needs to point at a space that is *above* human height while seated and within human height when standing.

### 3.5. Data Analysis to Identify Events

The proposed device collects the relevant data. The data are then analyzed to capture the events. The entire system is composed of two components:

- A device on the user’s desk with a local client software that collects the data;
- The cloud server where the data is processed.

Any event notification is sent back to the client eHealth software for prompting. The server performs the time series analysis on the data. In the proposed system, we only aim to capture selected events (Table 2):

The data analysis happens in real time in the cloud. This occurs in a window of time equivalent to the time taken by an employee to sit down or stand up, *δ*. Typically, this is in a few hundred milliseconds for healthy, adult human beings. In the time period *δ*, the set of time series points that define a sit-down and stand-up event is signified by, *y_b_*, based on data from the Sensor *B*. The set of current, incoming set of distances for sensors *A* and *B* are represented by $x_{a}^{\delta }$ and $x_{b}^{\delta }$, respectively. The sizes of $x_{a}^{\delta }$ and $x_{b}^{\delta }$ are limited by *δ*. This effectively measures the similarity between the expected pattern, *y_b_,* and the current distance value, $x_{b}^{\delta },\;$in the time window *δ*. If the similarity distance is less than a threshold *α*, then it is considered to be a match. The value of *α* depends on the actual environment of the employee—the height of the person, the distance between the monitor and the person, and the angles at which the sensors are pointing.

The $x_{a}^{\delta }\;$is monitored for values below or above *β*, which signify if the user has been detected by Sensor *A*. The value of the $x_{a}^{\delta }$ is normalized between 1 and 0, where 1 represents the maximum expected value of the distance between Sensor *A* and the person. Any value over this distance is ignored and considered to be a distance of 1 in the normalized form. This is to ensure that only the target user is observed and that no other person behind the target user is selected. The algorithm to determine the event in real time is as follows (Algorithm 1):
**Algorithm 1** Cloud-based Data Analysis*Inputs: y_a_*, *y_b_*, *α*, *β*, $x_{a}^{\delta }$, $x_{b}^{\delta }$ *Output: Event code* (0, 1, 2) 
Normalize all the time series so that all time series *y* and *x_b_* have a value between 0 and 1, inclusive.Normalize *x_a_* based on the maximum distance expected between the sensor and the user.Calculate DTW alignment cost between *y_b_*, *x_b_*event = 0if *cost*(*y_b_*, ) *< α* and $\bar{\;x_{a}^{\delta }}$ *< β*, then event = 1else if *cost*(*y_b_*, $x_{b}^{\delta }$) *< α* and $\bar{x_{a}^{\delta }}$ *> β*, then event = 2

The client software aims to ensure that the user/employee has made a stand-up and sit-down transition at least once every few hours (*T*). This is a user-related setting set by the user as per their desires. It has bounded limits for health benefits. The client software receives this event code in real time. It stores the value of an event over a period of time. The client software works by using a timer to keep track of actions and prompts. If the cloud confirms that the stand-up and sit-down actions have taken place, then the client does not prompt the user and simply records the activity and resets the timer. If the timer reaches a value of *T* and no confirmation is received, this means that the user has been sitting for the entire duration. In this case, the user is prompted to take the action of standing up and sitting down. The device then observes this, records it, and resets the timer.

### 3.6. Integration with the eHealth System

**Figure 4:**
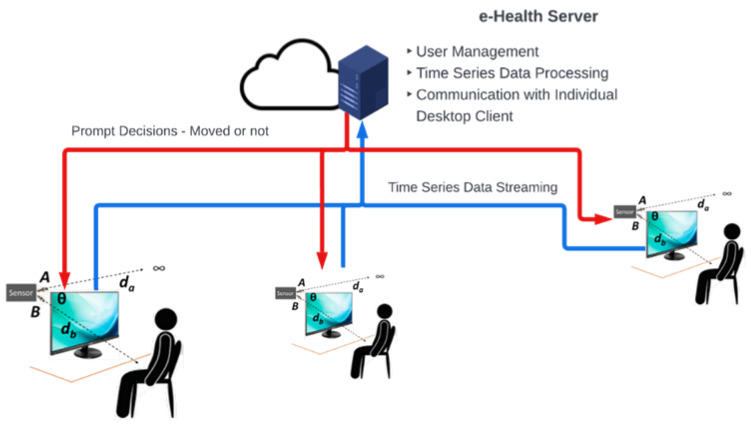
The cloud-based eHealth architecture with multiple users.

Figure 4 shows the IoT-based, smart office eHealth system architecture. As described above, the office has multiple desk sensors. The sensors each have a unique ID number. While the sensor can be associated with a desk, it does not identify a person. Each sensor streams the two values for *d*_a_ and *d_b_* to a a client software installed on the computer. The communication is through USB, which also powers the device. The client software is responsible for prompting the users to move and keeps track of their movements (e.g., sit–stand transitions). It also sends the streaming data to the cloud after cleansing the data with smoothing average techniques.

The eHealth system provides user management features. It stores the following information:

- Number of times the user is expected to move in a day;
- Working hours of the employee;
- Specific values of *T*, *δ* and *α* for the user and desk.

## 4. Implementations and Results

### 4.1. The Implementation

**Figure 5:**
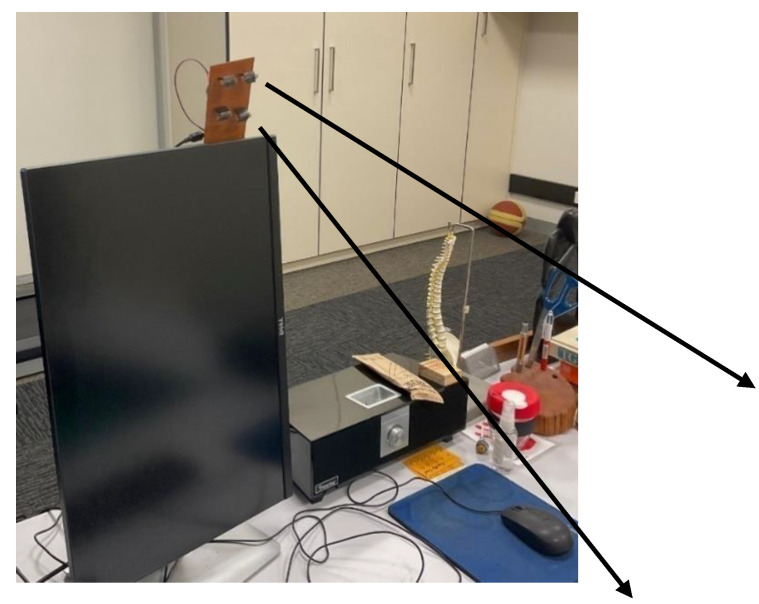
The prototype device deployed on a desk. The two arrows show the orientation of the sensor towards the position of the person.

The implementation is shown in Figure 5. We used multiple VL53L0X sensors. We used two sets of sensors for A and B. Both sets return the same information and are redundant; however, they were utilized to ensure that appropriate, error-free data are collected for analysis. The device is clamped to the monitor as described in Section 3. An Arduino Nano is used as the microcontroller, which is cabled to the PC as a USB device.

The client software is written in Java and uses Serial communication libraries to collect data from the microcontroller. The client software is connected to the cloud via WebSocket to stream data in real time. The client-side analysis software is written in Python using the required libraries for DTW. DTW is the time series similarity algorithm used for this implementation.

The prototype device (Figure 5) uses sensors less than 1 cm (about 0.39 in) in each dimension. With proper encasing, the device will be small and inconspicuous to the user.

### 4.2. Testing and Results

The device was tested with a person performing sit–stand transition movements in front of it. The exact movements were recorded manually to establish the ground truth. We tested the device with the following parameters (Table 3):

The first four parameters are static and relate to the person whose data was captured for analysis in this paper. The sampling rate was changed to observe any drop in the accuracy and timing of detection. The values of the final two parameters are the best values, which were found to work to capture the most accurate data for the given person.

In this section, we describe the process from the point of view of the sensor and the client software, which is responsible for generating the movement break prompts. We consider the data being collected in real time, uploaded to the server, and the event codes received in real time.

**Figure 6:**
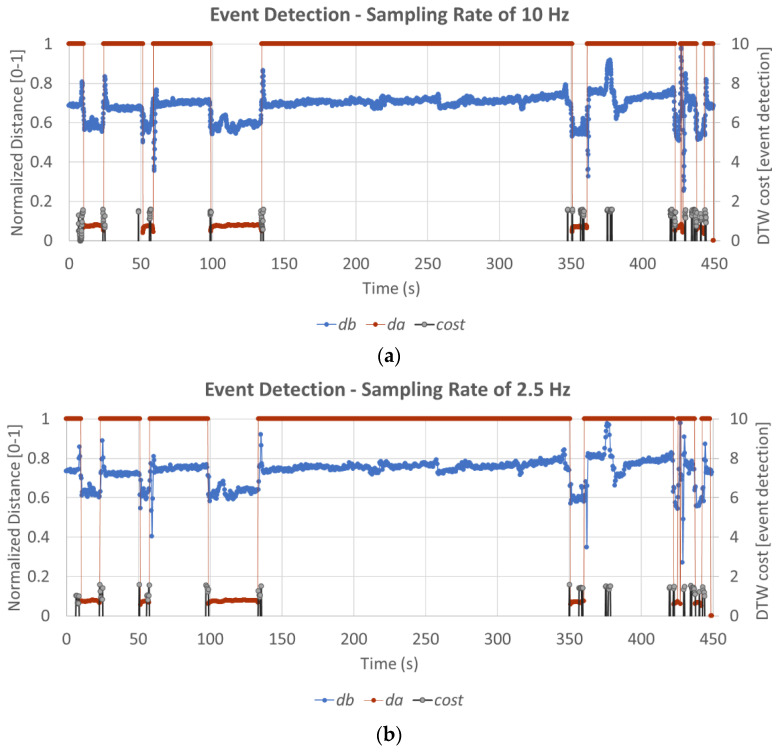
The time series analysis to identify the sit-down and stand-up actions at (**a**) 10 Hz and (**b**) 2.5 Hz. For the *cost*(*y_b_*, $x_{b}^{\delta }$), only the values less than 0.2 are plotted; the values above 0.2 are ignored for easy visual understanding.

Figure 6 shows the result of the data analysis algorithm for the detection of events. The data from the two sensors are shown along with the *cost*(*y_b_*, $x_{b}^{\delta }$), calculated in real time. The calculated DTW cost function value is less than α when the movements occur. All cost values above α are ignored.

Furthermore, the cluster of bursts of the cost values can be tallied with the value of the corresponding $x_{a}^{\delta }$. From the Algorithm 1’s lines 5 and 7, if within the *lag*, the client software detects $x_{a}^{\delta }$ to be low as well, and the stand-up is confirmed. This happens multiple times in Figure 6 before 150 s.

However, if the $x_{a}^{\delta }$ is high, then some movement was detected by Sensor *B* that is similar to standing up, but no change was observed by Sensor *A*. This means the whole action is not confirmed. This happens at time 370 s in the chart below. The detection of the action is less deterministic when there is a massive, quick change in the value of *d_b_* and *d_a_*.

**Figure 7:**
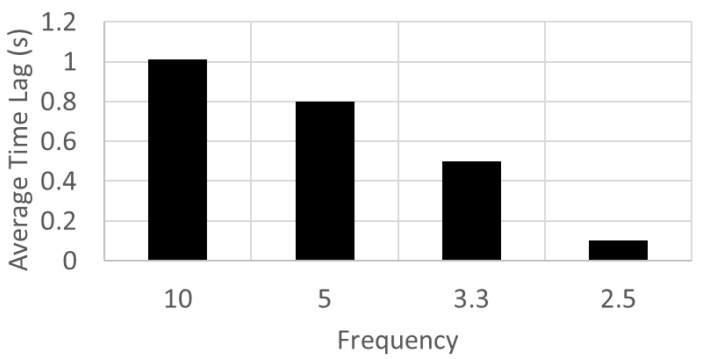
The average time difference for different frequencies of data collection.

Changing the sampling rate slightly alters the point of time at which the events are detected relative to the actual movement time. In fact, the algorithm detects the movement before the full movement occurs when measured at 10 Hz, most likely due to environmental noise. Figure 6b shows the detection of events with the same data set at 2.5 Hz. Most of the detection events occur at the same time. However, the temporal alignment of the detection with the drop in the value of *d_a_* is much more accurate in when sampling at 2.5 Hz compared to sampling at 10 Hz. This is seen around the 50 s time in Figure 6. This denotes a difference in time of detection. Figure 7 shows the absolute average difference in time of detection for different frequencies.

Thus, even though data is collected and processed at high speed, the desktop eHealth client software needs to work with a slight *lag* (e.g., 1 min) in processing the appropriate cost values. There are multiple instances of the cost values in short bursts. These short bursts are mostly times aligned with a drop in the Sensor *A* value and changes in Sensor *B*. If the client software observes data from the last 1 min and clusters them, it can identify that stand-up and sit-down action has occurred in the previous minute.

The proposed sensor meets all the criteria of the privacy-preserving feature triad. It collects *precise information* as it defines movement as two measurable physical distances only. Each desk in the office can be equipped with a unique sensor that can *trace* the performance of the desk’s occupant employee/worker over time. By selecting a specific value for *β*, it can ensure that only the occupant of the desk is observed. Any person standing just behind the seated person will not be observed as they will have a higher distance of *d_a_* than *β*. This ensures *isolated event* detection for the same person.

## 5. Results Discussion and System Characteristics

The proposed system has the following characteristics:

*i*. There is a possibility of a time gap between the matching of Sensors *A* and *B*. This is due to the speed at which the user moves at different times of the day. This difference may be used to identify additional employee parameters, such as stress or fatigue;
*ii*. This sensory array can detect the on-desk behaviors of sitting down and standing up during work with high accuracy of over 90%. The accuracy improves for longer time gaps (e.g., 5 s for the presented data set). Moreover, in the context of this application, if the device is unsure of the actions, it can ask the user to perform the action and monitor the action upon prompt;
*iii*. The non-wearable sensor design does not have limitations with respect to power. The microcontroller in the device can connect to the computer as a peripheral device with a USB cable. The power consumption requirement is not high and can be achieved with 3.3 V distance sensors, consuming less than 10 mA with efficient manufacture;
*iv*. This device is low-cost and has a suitable selection of raw components;
*v*. This device can be also deployed per person in the office environment to provide individualized feedback. This allows for the personalized measurement of on-desk behaviors;
*vi*. The software client takes less than 1% of the CPU as a background process, placing no extra load on the employees’ PCs. Although inefficient, it is possible to package all the analyses within the client software, if needed. In that case, running the client on a PC can consume more than 1% of the CPU (depending on the capacity of the PC) to analyze real-time data locally;
*vii*. The proposed architecture has real-time streaming. If this streaming is disrupted, then the detection may have delays or be lost altogether. However, assuming the streaming always works, it requires an extremely low bandwidth of a few bytes per second.

## 6. Future Work

The proposed system, including the sensor and its use in eHealth, can be enhanced in many ways.

### 6.1. Future Sensor Integration in eHealth Applications

The non-wearable sensor device proposed in this paper can detect the on-desk behaviors of seated employees, providing a valuable tool for field-based researchers interested in measuring the health outcomes of workplace health interventions that target sedentary behavior. Addressing the limitations of wearable devices to measure desk behaviors, this non-wearable sensor array has no power or storage limitations, has a low cost, and does not require the employee to remember to wear the device. Moreover, wearable solutions present several barriers to researchers regarding employee recruitment and retention [62]. Appropriately placed, non-wearable sensors that measure desk behavior may allow health researchers to improve their sample recruitment numbers without sacrificing their analytical capabilities. We propose that non-wearable sensors are a better option to remain inconspicuous during field-based data collection, allowing health researchers to collect valid behavioral data during work hours.

Whilst the main aim of this device was to measure the on-desk behaviors of a seated employee, other parameters, such as fatigue and stress, may also be detected automatically and in real time. The privacy aspect of such detections and monitoring needs to be addressed separately.

### 6.2. Future Sensor Solution Developments

The proposed data analysis can detect the standing-up and sit-down actions of a seated employee. It can therefore be deployed ubiquitously in an office with multiple employees. Several factors determine the accuracy of the detection of the event, such as *δ*, *α*, *β*, *time lag*, and *θ.* The values for each of these are closely tied to the human and physical environment, e.g., desk height and sensor placement. The process for identifying these values can be improved.

In this paper, we proposed to identify only two actions. This is sufficient for the purposes of well-timed prompting to alter human sedentary behavior. However, it is also possible to identify more actions than just standing up and sitting down, including the engagement in common exercises within the office such as squats or running in place. A range of other aspects, such as fatigue and stress, may also be detected automatically and in real time. The privacy aspect of such detections and monitoring needs to be addressed separately.

## 7. Conclusions

This paper has proposed a new sensor design for tracking human movement and sedentary behavior in office environments. The system described herein aims to reliably detect the movement of a person seated at a desk. The movements include standing up from a seated position, potentially followed by a time when the person is away from the desk, returning to the desk, and retaking the seat. The assumption is that workers will have their desks, monitor, and computer within a typical office environment. Once the sensor and the client software on their PCs detect the movement, it records the event in the eHealth system. With the use of such a sensor, eHealth systems can more reliably capture human actions based on data captured via the non-wearable sensor. We have shown that this device can detect these actions with a proper time series analysis and may be improved to capture other actions as well. With its privacy-preserving features, it can be widely adopted in various environments.

## Figures and Tables

**Table 1:** Comparison of Sensor technologies.

	*Accelerometer* [38,39,40]	*Camera* [59]	*PIR* [60]	*Pressure Sensor* [61]	*Sound Sensor* [49,50,51]
***Precise Information***	Medium: Can identify individual identity.	Low: Can identify individual identity and record content.	High: Unobtrusive.	Medium: Can identify individual identity.	Low: Can identify individual identity and record content.
***Traceable***	Medium: Tracks movement information.	High: Tracks presence, identity, location information.	Medium: Tracks presence information.	Medium: Tracks presence, identity information.	Medium: Tracks presence, identity information.
***Isolated Event Detection***	High: Data binding to individual identity.	High: Accurately identifies individual data.	Low: Cannot isolate individuals from the group.	High: Data binding to individual identity.	Medium: Depends on feature extraction.
***Security***	High: Local data process.	Low: Cloud data process.	High: Local data process.	High: Local data process.	Low: Cloud data process.
***Comfort***	Low: Must attach to body physically all the time.	High: Non-intrusive, unobtrusive.	High: Non-intrusive, unobtrusive.	Medium: Attached to body, unobtrusive.	High: Non-intrusive, unobtrusive.
***Cost***	Medium	High	Low	Low	Low
***Energy***	Medium energy consumption. Attaches to a small battery.	High energy consumption. Attaches to a small battery.	Medium energy consumption. Attaches to a stable power source.	Low energy consumption. Attaches to a large battery.	Medium energy consumption. Attaches to a stable power source.

**Table 2:** Event Codes.

Event Code	Event
0	Sitting position
1	Standing up
2	Unknown

**Table 3:** Test Parameters.

	Parameter	Value
1	Approximate sitting down and standing up time (*δ*)	300 ms
2	Height of the person (standing)	183 cm
3	Height of the person (sitting)	120 cm
4	Dataset size	4500 points (450 s)
5	Sampling rate	10 Hz, 5 Hz, 3.3 Hz, and 2.5 Hz
6	Rolling average window size	4
7	α DTW cost function threshold	1.6
8	β individual distance threshold	0.2

## Data Availability

Data sharing not applicable.
